# Outcomes Following a Mental Health Care Intervention for Children in the Emergency Department: A Nonrandomized Clinical Trial

**DOI:** 10.1001/jamanetworkopen.2024.61972

**Published:** 2025-02-26

**Authors:** Amanda S. Newton, Jennifer Thull-Freedman, Jianling Xie, Teresa Lightbody, Jennifer Woods, Antonia Stang, Kathleen Winston, Jacinda Larson, Bruce Wright, Michael Stubbs, Matthew Morrissette, Stephen B. Freedman

**Affiliations:** 1 Department of Pediatrics, Faculty of Medicine and Dentistry, University of Alberta, Edmonton, Alberta, Canada; 2 Departments of Pediatrics and Emergency Medicine, Cumming School of Medicine, University of Calgary, Calgary, Alberta, Canada; 3 Departments of Pediatrics, Cumming School of Medicine, University of Calgary, Calgary, Alberta, Canada; 4 Children, Youth, and Families—Addictions and Mental Health, Alberta Health Services, Edmonton, Alberta, Canada; 5 University of Alberta Hospital and Stollery Children’s Hospital Emergency Departments, Edmonton, Alberta, Canada; 6 Alberta Children’s Hospital Research Institute, University of Calgary, Calgary, Alberta, Canada; 7 Department of Psychiatry, Cumming School of Medicine, University of Calgary, Calgary, Alberta, Canada; 8 Department of Psychiatry, Faculty of Medicine and Dentistry, University of Alberta, Edmonton, Alberta, Canada; 9 Alberta Children’s Hospital Foundation, Calgary, Alberta, Canada

## Abstract

**Importance:**

The emergency department (ED) is an important safety net for children experiencing mental and behavioral health crises and can serve as a navigational hub for families seeking support for these concerns.

**Objectives:**

To evaluate the outcomes of a novel mental health care bundle on child well-being, satisfaction with care, and health system metrics.

**Design, Setting, and Participants:**

Nonrandomized trial of 2 pediatric EDs in Alberta, Canada. Children younger than 18 years with mental and behavioral health presentations were enrolled before implementation (preimplementation: January 2020 to January 2021), at implementation onset (run-in: February 2021 to June 2021), and during bundle delivery (implementation: July 2021 to June 2022).

**Intervention:**

The bundle involved risk stratification, standardized mental health assessments, and provision of an urgent follow-up appointment after the visit, if required.

**Main Outcomes and Measures:**

The primary outcome, child well-being 30 days after the ED visit, was assessed using the Stirling Children’s Wellbeing Scale (children aged <14 years) or Warwick-Edinburgh Mental Wellbeing Scale (children aged 14-17 years). Change in well-being between the preimplementation and implementation periods was examined using interrupted time-series analysis and multivariable modeling. Changes in health system metrics (hospitalization, ED length of stay [LOS], and revisits) and care satisfaction were also examined.

**Results:**

A total of 1412 patients (median [IQR] age, 13 [11-15] years), with 715 enrolled preimplementation (390 [54.5%] female; 55 [7.7%] First Nations, Inuit, or Métis; 46 [6.4%] South, Southcentral, or Southeast Asian; and 501 [70.1%] White) and 697 enrolled at implementation (357 [51.2%] female; 51 [7.3%] First Nations, Inuit, or Métis; 39 [5.6%] South, Southcentral, or Southeast Asian; and 511 [73.3%] White) were included in the analysis. There were no differences between study periods in well-being. Reduced well-being *z* scores were associated with mood disorder diagnosis (standardized mean difference, −0.14; 95% CI, −0.26 to −0.02) and nonbinary gender identity (standardized mean difference, −0.41; 95% CI, −0.62 to −0.19). The implementation period involved fewer hospitalizations (difference in hospitalizations, −6.9; 95% CI, −10.4 to −3.4) and longer ED LOS (1.1 hours; 95% CI, 0.7 to 1.4 hours). There were no differences between study periods in ED revisits or care satisfaction.

**Conclusions and Relevance:**

In this study, the delivery of a care bundle was not associated with higher child well-being 30 days after an ED visit. Hospitalizations did decrease during bundle delivery, but ED LOS did not. These health system findings may have been affected by broader changes in patient volumes and flow processes that occurred during the COVID-19 pandemic, which took place as the study was conducted.

**Trial Registration:**

ClinicalTrials.gov Identifier: NCT04292379

## Key Points

**Question:**

Compared with standard care, does implementing an evidence-based mental health care bundle in the emergency department (ED) improve child well-being 30 days after an ED visit?

**Findings:**

In this nonrandomized trial of 1412 patients, there were no differences between the standard care and mental health care bundle delivery periods for child well-being 30 days after the ED visit.

**Meaning:**

These findings suggest that a novel care bundle for patients experiencing mental and behavioral health crises was not associated with higher child well-being after the ED visit.

## Teaser

This nonrandomized clinical trial evaluates the outcomes of a novel mental health care bundle in the emergency department (ED) on child well-being, satisfaction with care, and health system metrics.

## Introduction

The emergency department (ED) is a safety net for children experiencing mental and behavioral health crises.^1^ Reasons for ED visits vary but commonly relate to suicidal ideation, depressed or anxious mood states, and aggressive behavior.^2,3,4^ Crises can also involve medical and social concerns, which can exacerbate presenting complaints. To care for this diverse patient population, ED clinicians need a range of resources and skills.^5^

The American Academy of Pediatrics (AAP) recently reaffirmed the importance of providing optimal and equitable mental health care in EDs.^6,7^ Recommendations to achieve this care include deploying strategies and resources to assist clinicians in assessing needs and providing initial care as well as establishing consultation and referral pathways. Multiple studies point to clinical practice variation across settings,^8,9,10,11,12,13^ making initiatives to standardize and improve care important.

We evaluated the outcomes of an ED care bundle for children presenting with mental and behavioral health concerns. The care bundle was aligned with AAP recommendations^6,7^ and involved collectively deploying a set of evidence-based practices.^14^ Our primary objective was to evaluate the bundle’s association with child well-being 30 days after an ED visit. Secondary objectives included examining care satisfaction and health system metrics outcomes. We hypothesized that the care bundle would improve child well-being through clinician use of mental health tools and booking follow-up care and lead to care efficiencies that would result in increased care satisfaction and reduced length of stay (LOS), hospitalizations, and return ED visits.

## Methods

### Design and Setting

This nonrandomized trial took place in 2 pediatric EDs in Alberta, Canada, and was conducted using a time-series design in 3 periods: (1) preimplementation, with no changes to care (January 29, 2020, to January 31, 2021) and baseline data collected for 12 months; (2) run-in, where care bundle components were introduced (February 1, 2021, to June 30, 2021); and (3) implementation, where the bundle was fully integrated into care (July 1, 2021, to June 30, 2022) and data were collected for another 12 months. Patient engagement^15,16^ involved codesigning the care bundle’s purpose, reviewing and coapproving the bundle’s components, coselecting study outcomes and measures, and longitudinal consultation during study conduct (eg, refining clinical processes that supported bundle delivery). The study was approved by site research ethics boards. Caregivers consented to study participation; assent or consent was obtained from children as appropriate. The protocol (see Supplement 1) was registered before participant enrollment and published.^17^ We report results in accordance with the Transparent Reporting of Evaluations With Nonrandomized Designs (TREND) reporting guideline.^18^

### Population

We enrolled children younger than 18 years presenting with the concerns in the Canadian Emergency Department Information System: anxiety, bizarre behavior, concern for patient’s welfare, depression, disruptive behavior, insomnia (secondary to anxiety or worries), nonsuicidal self-injury, suicidal or homicidal ideation, situational crisis, or violent behavior. Children were ineligible if they required medical treatment or stabilization before mental health care (eg, for hallucinations or self-harm injuries) which included being brought to the ED by police or peace officers (who support work of police). Children were also ineligible if there was a communication barrier at triage or they previously participated in the study.

### Interventions

#### Standard Care

Before the study and during the preimplementation period, standard care did not include the use of specialized mental health tools during triage or bedside care and consisted of ED physicians overseeing access to psychiatric consultation. Following discharge, families were responsible for organizing their child’s follow-up care (eFigure 1 in Supplement 2).

#### Mental Health Care Bundle

The bundle standardized suicide risk screening^19^ at triage, a focused and collaborative mental health assessment,^20^ and a follow-up appointment booking process, and introduced 2 practices not commonplace in pediatric EDs: preference-based flow at triage based on the screening outcome^21^ and direct connection to follow-up. The bundle was developed to be the new standard of care for patients with eligible mental health concerns. Given this, no blinding took place during the study.

#### Suicide Risk Screening

Triage nurses screened children aged 8 years or older for suicide risk using the Ask Suicide-Screening Questions (ASQ)^22,23^ to identify high-risk patients and initiate safety protocols when necessary.^11^ Children who answered yes to any ASQ question, were unable to or refused to answer, or were younger than the recommended screening age (<8 years) received an ED evaluation. Children who answered no to all questions and did not report any medical or safety concerns were eligible to have a care navigation conversation with a health care provider that outlined the option of accepting a scheduled, urgent appointment at a mental health clinic in lieu of remaining in the ED for care.

#### Focused, Collaborative Mental Health Assessment

Children admitted to the ED, regardless of screening result, were assessed using the HEADS-ED, a mnemonic-based tool with 7 scored domains (home, education, activities/peers, drug/alcohol, suicidality, emotions and behavior, discharge resources).^20,24^ The tool was completed by the first available health care provider, either a mental health nurse or ED physician. Domain scores (0 = no/minimal impairment, no action needed; 1 = moderate impairment, needs action but not immediate; or 2 = severe impairment, needs immediate action) were totaled. Health care providers were advised to use a total score of 8 and above or a suicidality domain score of 2 to identify patients needing psychiatric consultation; this score has previously been demonstrated to be associated with mental health consultation and hospital admission.^20^ Patients with lower scores than this could be discharged if consistent with the clinical judgment of the attending ED physician. When the assessment was conducted by a mental health nurse, the nurse reviewed the patient’s case with an ED physician before disposition. Medical clearance was not required; additional assessments were conducted at the physician’s discretion based on information collected during the assessment. This approach is supported by evidence^25^ and the Choosing Wisely campaign.^26^

#### Urgent Appointment

The scheduled urgent appointment had 2 purposes: (1) increasing the likelihood of receiving follow-up by removing access barriers,^27^ and (2) introducing preference-based care at triage, an approach that is safe and effective for select conditions.^28,29^ Appointments were available at triage to low-risk patients and at discharge for those without a mental health care provider to provide follow-up. Appointments took place at a partnered mental health clinic, ideally within 96 hours of the ED visit, and were modeled after choice from the Choice and Partnership Approach,^30^ which promotes collaborative mental health care planning.^31^ Appointments were intended to be 60 minutes in duration.

### Bundle Implementation

During the run-in period, health care providers received resources (eg, pocket cards for quick reference) and an in-service training session relevant to their role led by a nurse educator (ASQ) or physician (HEADS-ED) or mental health lead (urgent follow-up). Clinical leaders introduced change management strategies while overseeing workflow optimization. Visual diagrams and plan-do-study-act cycles were used to test changes before full implementation, and run charts were used to measure and improve adoption of bundle elements.^32^

### Data Collection

Patient-reported data were collected via telephone or online through REDCap.^33^ Caregivers and participants (based on age) completed surveys following the ED visit (baseline), at 30 days, and at 3- and 6-month follow-ups. Nonrespondents received up to 4 email reminders followed by a telephone call. Data related to participants’ ED visits were collected by research staff through medical record review. Sociodemographic characteristics including gender identity and ethnic and racial background were self-reported by participants or caregivers at baseline. Because some individuals described their racial identity in an open text field for ethnicity reporting, we revised the characteristic to include both ethnic and racial identity. Data on ED volumes for all mental health visits and LOS for all visits were obtained from administrative sources.

### Study Outcomes

The primary outcome, child well-being 30 days after the index ED visit, was chosen by patient-partners as it reflects important improvements in comfort, health, and happiness. It was measured using the Stirling Children’s Wellbeing Scale^34,35^ with children younger than 14 years (internal consistency, Cronbach α = 0.85; test-retest reliability,* r* = 0.75) or the Warwick-Edinburgh Mental Wellbeing Scale^36^ with children 14 years and older (internal consistency, Cronbach α = 0.89; test-retest reliability, *r* = 0.83). For both scales, higher scores represent higher well-being.

Secondary outcomes were child well-being at 3- and 6-month follow-up, care satisfaction measured at baseline using the Service Satisfaction Scale^37^ (caregivers, 15 items; children, 13 items), index visit LOS (time interval between triage and discharge) and hospitalization, and unscheduled ED revisits for mental health care within 72 hours and 30 days (potential unintended effects).

### Statistical Analysis

An intention-to-treat approach was used for analysis. For the primary analysis, we used a segmented linear regression to obtain level and slope changes using the mean well-being scores for each month before and after bundle implementation; separate analyses were conducted for each well-being measure.^38^ In this model, a level change represents the estimated effect of the bundle shortly after its implementation, and a slope change represents this effect over time. To explore patient-level associations, a regression model was used and included random intercept terms for study site and participant and a random slope term for well-being assessment time points. In this model, data were standardized across age groups using *z* scores to derive a single measure of well-being. The model was adjusted for patient age, gender, ethnic and racial background, discharge diagnosis, assessment time point, study period (preimplementation or implementation), and visit acuity (triage score^39^). Interactions between study period and assessment time points were included to assess intervention effect over time.

Multivariable linear regression models, fitted with generalized estimating equations, were used to assess associations between bundle use with ED LOS and care satisfaction. The model was adjusted for patient age, gender, ethnic and racial background, visit acuity, discharge diagnosis, type of mental health care received (ED mental health care provider, child and adolescent psychiatrist), index visit hospitalization, and study period. To account for the COVID-19 pandemic during the study, for each study site, the model included the monthly number of mental health visits and median ED LOS across all mental health visits. Interaction terms were added to account for changes to ED function and flow that occurred during the pandemic. In the ED LOS model, we used the natural log transformed hours. In the satisfaction model, we used *z* scores to derive a single measure of satisfaction. The proportion of children with index visit hospitalizations and unscheduled ED revisits were compared using χ^2^ or Fisher exact tests.

Before study launch, we estimated that during each interrupted time series analysis period (month) we would enroll 50 or more participants.^40^ We used SPSS version 29.0 (IBM) and R version 4.3 (R Project for Statistical Computing) for all analyses. Statistical significance was defined as *P* < .05 and all analyses were 2-tailed.

## Results

A total of 1412 patients (median [IQR] age, 13 [11-15] years), with 715 enrolled preimplementation (390 [54.5%] female; 55 [7.7%] First Nations, Inuit, or Métis; 46 [6.4%] South, Southcentral, or Southeast Asian; and 501 [70.1%] White) and 697 enrolled during implementation (357 [51.2%] female; 51 [7.3%] First Nations, Inuit, or Métis; 39 [5.6%] South, Southcentral, or Southeast Asian; and 511 [73.3%] White) were included in the analysis (Figure). Most participants were receiving mental health care before their ED visit; Table 1. A summary of baseline well-being scores by age and across study periods is presented in eTable 1 in Supplement 2.

**Figure.**
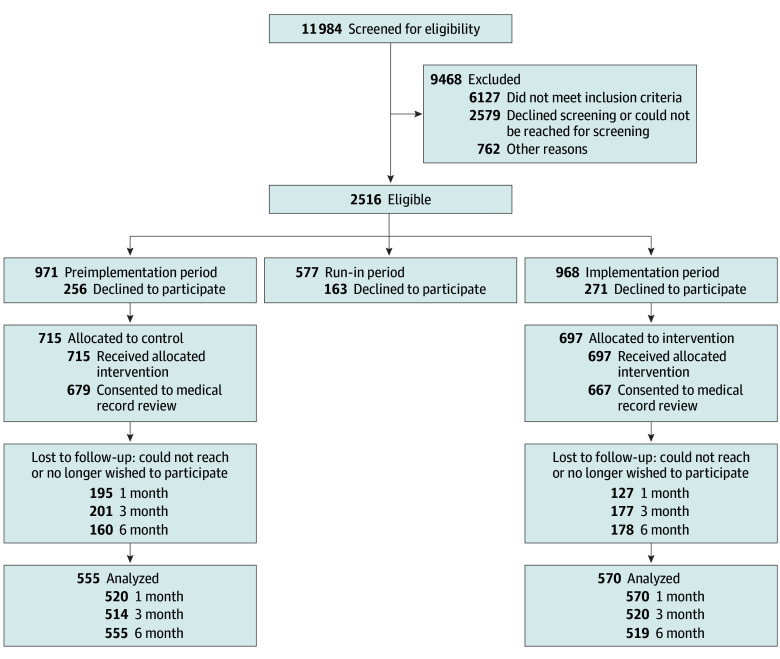
Flow of Participants Through the Study

**Table 1.** Participant Sociodemographic and Clinical Characteristics During the Study Periods

Characteristic	Participants, No. (%)	
	Preimplementation	Implementation
Sociodemographic		
Total patients	715	697
Age, y		
<14	397 (55.5)	348 (49.9)
≥14	318 (44.5)	349 (50.1)
Gender identity		
Female	390 (54.5)	357 (51.2)
Male	270 (37.8)	254 (36.4)
Nonbinary^a^	19 (2.7)	45 (6.5)
Transgender^b^	19 (2.7)	22 (3.2)
Declined to answer or missing	17 (2.4)	19 (2.7)
Ethnic and racial background^c^		
Black	14 (2.0)	13 (1.9)
First Nations, Inuit, or Métis	55 (7.7)	51 (7.3)
Latin American	13 (1.8)	14 (2.0)
South, Southcentral, or Southeast Asian	46 (6.4)	39 (5.6)
West Asian	2 (0.3)	0
White	501 (70.1)	511 (73.3)
Multiple groups^d^	64 (9.0)	50 (7.2)
Indeterminate answer	5 (0.7)	8 (1.1)
Declined to answer or missing	15 (2.1)	11 (1.6)
Median household total income, CAD $^e^		
100 000-999 999	132 (18.5)	172 (24.7)
90 000-99 999	542 (75.8)	506 (72.6)
80 000-89 999	23 (3.2)	3 (0.4)
<79 999	6 (0.8)	7 (1.0)
Missing	12 (1.7)	9 (1.3)
Community size of residence		
Large (≥100 000)	539 (75.4)	502 (72.0)
Medium (30 000-99 999)	94 (13.1)	100 (14.3)
Small (10 000-29 999)	54 (7.6)	77 (11.0)
Very small (<10 000)	16 (2.2)	9 (1.3)
Missing	12 (1.7)	9 (1.3)
Currently receiving mental health care		
Yes	578 (80.8)	563 (80.8)
No	124 (17.3)	124 (17.8)
Declined to answer	13 (1.8)	10 (1.4)
Index visit characteristic		
Total patients	679	667
Visit acuity per CTAS		
1: Resuscitation	0	1 (0.1)
2: Emergent	224 (33.0)	263 (39.4)
3: Urgent	354 (52.1)	332 (49.8)
4: Less urgent	99 (14.6)	68 (10.2)
5: Nonurgent	2 (0.3)	3 (0.4)
Diagnosis^f^		
Suicidal ideation	187 (27.5)	190 (28.5)
Neurotic, stress-related, or somatoform disorder	189 (27.8)	145 (21.7)
Mood disorder	168 (24.7)	154 (23.1)
Family functioning or psychosocial needs	143 (21.5)	158 (23.7)
Behavioral or emotional disorder	78 (11.5)	36 (5.4)
Other mental disorder^g^	52 (7.7)	48 (7.2)
Diagnosis not documented in medical record	25 (3.7)	56 (8.4)
Length of stay, median (IQR), h	4.0 (2.0-6.0)	5.0 (3.0-8.0)
Hospital admission at discharge		
Both study sites	109 (16.1)	61 (9.1)
Stollery Children’s Hospital (n = 224)	12 (5.4)	7 (2.6)
Alberta Children’s Hospital (n = 455)	97 (21.3)	54 (13.5)

Abbreviation: CTAS, Canadian Triage and Acuity Scale.

^a^ Agender, pangender, genderqueer, gender fluid, gender nonconforming, or in the process of deciding.

^b^ Transmale, transmasculine, transfemale, transfeminine, or transgender nonbinary.

^c^ Grouped using Statistics Canada categories^41^ for visible minorities to account for open-category responses.

^d^ Two or more of the ethnicities or identities already listed.

^e^ Calculated using self-reported residential postal code.^42^

^f^ Grouped using the *International Classification of Diseases, Tenth Revision*, diagnostic clusters.^43^

^g^ Self-harm not requiring medical care, disorder of personality or behavior, disorder of psychological development, mental or behavioral disorder due to substance use, or unspecified disorder.

### Child Well-Being

Child well-being scores were not higher following bundle implementation (Table 2; eFigure 2 in Supplement 2). The level change for children younger than 14 years was −1.98 (95% CI, −5.60 to 1.64) and the slope change was −0.32 (95% CI, −0.82 to 0.19). For children 14 years and older, the level and slope changes were 0.85 (95% CI, −2.78 to 4.48) and 0.06 (95% CI, −0.47 to 0.58), respectively. The effect of the bundle implementation on child well-being was significantly greater at the 6-month assessment compared with baseline (standardized mean difference, 0.15; 95% CI, 0.01 to 0.29; *P* = .03) (Table 3).

**Table 2.** Unadjusted Model Examining Bundle Association With Child Well-Being 30 Days After the Index Emergency Department Visit

Model	Coefficient (SE) [95% CI]	*P* value
Children aged <14 y		
Average score preimplementation	37.34 (1.36) [34.67 to 40.00]	<.001
Trend baseline	0.26 (0.17) [−0.07 to 0.60]	.13
Level change after implementation	−1.98 (1.85) [−5.60 to 1.64]	.28
Trend change after implementation	−0.32 (0.26) [−0.82 to 0.19]	.22
Children aged ≥14 y		
Average score preimplementation	39.86 (1.39) [37.14 to 42.58]	<.001
Trend baseline	−0.12 (0.19) [−0.49 to 0.25]	.51
Level change after implementation	0.85 (1.85) [−2.78 to 4.48]	.65
Trend change after implementation	0.057 (0.27) [−0.47 to 0.58]	.83

**Table 3.** Multivariable Regression to Explore Bundle Association With Child Well-Being

Variable	Child well-being, standardized mean difference (95% CI)^a^	*P* value
Age, per year older	0.002 (−0.02 to 0.02)	.80
Gender identity		
Female	[Reference]	NA
Male	0.11 (0.01 to 0.21)	.03
Nonbinary	−0.41 (−0.62 to −0.19)	<.001
Transgender	0.15 (−0.13 to 0.42)	.30
Not specified	−0.14 (−0.55 to 0.28)	.52
Ethnic and racial background		
Black, Latin American, or West Asian^b^	0.28 (0.05 to 0.52)	.02
First Nations, Inuit, or Métis	−0.004 (−0.19 to 0.17)	.96
South, Southcentral, or Southeast Asian	0.28 (0.09 to 0.48)	.01
White	[Reference]	NA
Multiple	0.07 (−0.11 to 0.24)	.46
Not specified	0.21 (−0.17 to 0.59)	.29
Visit acuity, per 1 CTAS category lower	0.11 (0.04 to 0.19)	.002
Diagnosis^c^		
Suicidal ideation	−0.10 (−0.21 to 0.02)	.11
Neurotic, stress-related, or somatoform disorder	0.10 (−0.02 to 0.22)	.12
Mood disorder	−0.14 (−0.26 to −0.02)	.02
Behavioral or emotional disorder	−0.03 (−0.21 to 0.17)	.78
Self-harm not requiring medical care	−0.10 (−0.33 to 0.14)	.43
Any other assigned diagnosis^b^	0.02 (−0.11 to 0.16)	.78
Timing of well-being assessment		
6 mo after visit	0.57 (0.47 to 0.67)	<.001
3 mo after visit	0.65 (0.56 to 0.75)	<.001
30 d after visit	0.35 (0.27 to 0.43)	<.001
Baseline	[Reference]	NA
Study period		
Implementation	−0.05 (−0.17 to 0.07)	.41
Preimplementation	[Reference]	NA
Interaction term		
30-d assessment × implementation	0.008 (−0.11 to 0.12)	.89
3-mo assessment × implementation	0.07 (−0.06 to 0.20)	.32
6-mo assessment × implementation	0.15 (0.01 to 0.29)	.03
Effect of bundle implementation on well-being at assessment time points		
Effect estimates comparing preimplementation and implementation		
Child well-being at baseline		
Implementation	−0.05 (−0.17 to 0.07)	.41
Preimplementation	[Reference]	NA
Child well-being 30 d after visit		
Implementation	−0.04 (−0.16 to 0.07)	.48
Preimplementation	[Reference]	NA
Child well-being 3 mo after visit		
Implementation	0.02 (−0.11 to 0.14)	.79
Preimplementation	[Reference]	NA
Child well-being 6 mo after visit		
Implementation	0.10 (−0.03 to 0.23)	.13
Preimplementation	[Reference]	NA
Effect of assessment time points within preimplementation and implementation periods		
Effect estimates comparing assessment timing in the preimplementation period		
Assessment 6 mo after visit	0.57 (0.47 to 0.67)	<.001
Assessment 3 mo after visit	0.66 (0.56 to 0.75)	<.001
Assessment 30 d after visit	0.35 (0.27 to 0.43)	<.001
Assessment at baseline	[Reference]	NA
Effect estimates comparing assessment timing in the implementation period		
Assessment 6 mo after visit	0.72 (0.62 to 0.82)	<.001
Assessment 3 mo after visit	0.72 (0.63 to 0.82)	<.001
Assessment 30 d after visit	0.36 (0.28 to 0.44)	<.001
Assessment at baseline	[Reference]	NA

Abbreviations: CTAS, Canadian Triage and Acuity Scale; NA, not applicable.

^a^ The dependent variable is a *z* score of child well-being; coefficients represent differences in SDs of the well-being score.

^b^ Groups combined due to sample size.

^c^ The reference group is without the individual specific diagnosis.

Several features were associated with increased well-being scores: identifying as male; being of Asian, Black, or Latin American ethnic and racial background; and a lower triage acuity (Table 3). A mood disorder diagnosis (standardized mean difference, −0.14; 95% CI, −0.26 to −0.02) and nonbinary gender identity (standardized mean difference, −0.41; 95% CI, −0.62 to −0.19) were associated with reduced scores. During the preimplementation and implementation periods, well-being scores increased across assessment time points with the largest increases occurring at 3- and 6-month follow-ups during bundle delivery (standardized mean difference at 3 months: 0.72; 95% CI, 0.63 to 0.82; at 6 months, 0.72; 95% CI, 0.62 to 0.82) (Table 3). In a post hoc analysis, we explored whether associations differed between preadolescents (<13 years) and adolescents (13-17 years); in these models (eTables 2 and 3 in Supplement 2), self-harm not requiring medical care was associated with reduced preadolescent well-being scores, while findings for adolescents were similar to the overall model.

### Health System Metrics

Fewer index visits resulted in hospitalization in the implementation period compared with the preimplementation period (difference, −6.9; 95% CI, −10.4 to −3.4) (Table 1). There were no differences between the preimplementation and implementation periods in the proportion of ED revisits within 72 hours (difference, 1.1%; 95% CI, −0.4% to 2.6%) or 30 days (difference, 2.4%; 95% CI, −0.7% to 5.5%).

Median (IQR) ED LOS was 4.4 (2.8-6.8) and 5.8 (3.6-8.7) hours during the preimplementation and implementation periods, respectively (difference, 1.1 hours; 95% CI, 0.7 to 1.4 hours). In the regression analysis, LOS was positively associated with patient age and ED visit features (higher triage acuity, visits for suicidal ideation, visit resulted in hospitalization, and seen by a mental health care provider), number of all monthly mental health visits, and median ED LOS for all patients (Table 4). Bundle implementation was associated with an LOS increase of 44.8% (95% CI, 32.3% to 56.8%) among participants enrolled at the Alberta Children’s Hospital only.

**Table 4.** Regression Model Examining Bundle Association With ED Length of Stay and Care Satisfaction

Variable	ED LOS^a^		Care satisfaction^b^	
	Adjusted mean difference in natural log (95%CI)	*P* value	Standardized mean difference (95%CI)	*P* value
Bundle implementation				
Implementation	0.03 (−0.08 to 0.15)	.55	−0.18 (−0.29 to −0.06)	.003
Preimplementation	[Reference]	NA	[Reference]	NA
Age, per year older	0.01 (0.0003 to 0.03)	.05	−0.01 (−0.03 to 0.02)	.56
Gender identity				
Female	[Reference]	NA	[Reference]	NA
Male	0.01 (−0.06 to 0.09)	.69	−0.02 (−0.14 to 0.10)	.76
Nonbinary	0.04 (−0.12 to 0.19)	.62	−0.01 (−0.28 to 0.26)	.93
Transgender	0.09 (−0.10 to 0.28)	.33	−0.10 (−0.40 to 0.20)	.51
Not specified	0.06 (−0.21 to 0.32)	.67	−0.35 (−0.72 to 0.02)	.06
Ethnic and racial background				
Black, Latin American, or West Asian^c^	−0.03 (−0.2 to 0.13)	.68	0.39 (0.12 to 0.66)	.01
First Nations, Inuit, or Métis	0.10 (−0.03 to 0.22)	.12	−0.11 (−0.33 to 0.11)	.31
South, Southcentral, or Southeast Asian	−0.01 (−0.14 to 0.13)	.89	0.34 (0.14 to 0.53)	.001
White	[Reference]	NA	[Reference]	NA
Multiple	0.10 (−0.02 to 0.22)	.11	0.05 (−0.14 to 0.23)	.61
Not specified	0.15 (−0.10 to 0.41)	.24	0.44 (−0.05 to 0.93)	.08
Diagnosis^d^				
Suicidal ideation	0.17 (0.08 to 0.25)	<.001	0.20 (0.05 to 0.35)	.01
Neurotic, stress-related, or somatoform disorder	0.06 (−0.03 to 0.14)	.21	0.23 (0.08 to 0.39)	.003
Mood disorder	0.07 (−0.02 to 0.16)	.13	0.22 (0.07 to 0.37)	.01
Behavioral or emotional disorder	0.10 (−0.03 to 0.24)	.12	0.01 (−0.21 to 0.22)	.94
Self-harm not requiring medical care	0.08 (−0.09 to 0.25)	.36	0.07 (−0.19 to 0.33)	.60
Any other assigned diagnosis	0.11 (0.02 to 0.20)	.02	0.14 (−0.03 to 0.30)	.10
Visit acuity, per 1 CTAS category higher	0.08 (0.03 to 0.13)	.002	−0.03 (−0.11 to 0.06)	.51
Admitted at the index ED visit				
Yes	0.54 (0.42 to 0.67)	<.001	0.33 (0.10 to 0.57)	.01
No	[Reference]	NA	[Reference]	NA
Median ED LOS for all patients (provincial, quarterly data), h	0.10 (0.01 to 0.19)	.03	NA	NA
Natural log of h of ED stay (per h longer)	NA	NA	−0.02 (−0.02 to −0.01)	<.001
Total No. of mental health ED visits of the mo^e^	0.17 (0.10 to 0.24)	<.001	−0.0001 (−0.001 to 0.001)	.90
Type of ED mental health care				
Seen by an ED mental health care provider	0.27 (0.20 to 0.35)	<.001	0.29 (0.16 to 0.41)	<.001
Seen by a child and adolescent psychiatrist	0.64 (0.54 to 0.74)	<.001	0.06 (−0.11 to 0.24)	.47
Study site				
Alberta Children’s Hospital ED	−0.09 (−0.21 to 0.03)	.13	0.14 (−0.01 to 0.29)	.07
Stollery Children’s Hospital ED	[Reference]	NA	[Reference]	NA
Interaction term				
Implementation period × Alberta Children’s Hospital ED	0.33 (0.2 to 0.47)	<.001	NS	NA
Effect of bundle implementation on ED LOS at study site				
At Alberta Children’s Hospital	NA	NA	NS	NA
Implementation	0.37 (0.28 to 0.45)	<.001	NS	NA
Preimplementation	[Reference]	NA	NS	NA
At Stollery Children’s Hospital	NA	NA	NS	NA
Implementation	0.03 (−0.08 to 0.15)	.55	NS	NA
Preimplementation	[Reference]	NA	NS	NA
Effect of study site on ED LOS within study periods				
Before bundle implementation	NA	NA	NS	NA
Alberta Children’s Hospital ED	−0.09 (−0.21 to 0.03)	.13	NS	NA
Stollery Children’s Hospital ED	[Reference]	NA	NS	NA
After bundle implementation	NA	NA	NS	NA
Alberta Children’s Hospital ED	0.24 (0.14 to 0.34)	<.001	NS	NA
Stollery Children’s Hospital ED	[Reference]	NA	NS	NA

Abbreviations: CTAS, Canadian Triage and Acuity Scale; ED, emergency department; LOS, length of stay, in hours; NA, not applicable; NS, the interaction was not significant, thus excluded from the final model.

^a^ Natural log of ED LOS in hours.

^b^ The dependent variable is a *z* score of care satisfaction scale scores; coefficients represent differences in SDs of satisfaction scale scores.

^c^ Groups combined due to sample size.

^d^ The reference group is without the individual specific diagnosis.

^e^ Per 100 increased.

### Care Satisfaction

Care satisfaction *z* scores were lower during the implementation period in reference to the preimplementation period (standardized mean difference, −0.18; 95% CI, −0.29 to −0.06) (Table 4). Higher satisfaction was associated with ethnic and racial identity, visits for suicidal ideation, anxiety or mood disorders, assessment by an ED mental health care provider, hospitalization, and study site; reduced satisfaction was associated with increased LOS (Table 4).

## Discussion

In this study, we introduced a mental health care bundle in 2 pediatric EDs. Although this bundle was not associated with higher child well-being after the ED visit or a decrease in ED LOS, well-being was higher at 6-month follow-up and fewer hospital admissions occurred during bundle delivery.

In both study periods, the average well-being scores reported by younger children were 17% below the scores described in the Stirling Children’s Wellbeing Scale standardization study,^35^ while older children reported scores classified as being in the bottom 15% of scores for the Warwick-Edinburgh Mental Wellbeing Scale.^44^ Low participant scores may reflect experiences during the COVID-19 pandemic, including worsened familial relationships,^45,46,47^ social isolation,^46^ and/or increased mental health symptoms.^45,46,48^ In 1 pandemic-related study,^49^ caregivers who reported a greater impact from the pandemic reported more behavioral difficulties in their children. In this study,^49^ caregivers’ stress mediated conflicts with their children. In another study,^46^ improved caregiver-child relationships during the pandemic were associated with higher child well-being. These findings, alongside our findings of associations between child sociodemographics and well-being, suggest additional studies would be beneficial to (1) identify the complex and interacting needs of children who visit the ED with mental and behavioral health crises, and (2) inform specific foci for ED-based assessments and urgent follow-up appointments that could improve patient outcomes after bundle receipt.

Despite lack of change in well-being 1 month after the visit, our analysis indicated greater increases in 3- and 6-month well-being scores during bundle implementation compared with the preimplementation period. This delayed effect may be associated with care initiated by the urgent follow-up appointment or through an existing mental health care provider, which most children in our study had. In a recent large-scale US study,^50^ outpatient care within 1 month of an ED mental or behavioral health visit occurred for 55.8% of children, and additional outpatient visits took place between 31 days and 6 months after the ED visit (median [IQR], 6 [3-16] visits). In our study, we did not collect information on community-based mental health care service use during the 6-month postvisit period, which would have allowed us to identify differences between the study periods and among users and explore the impact on child well-being.

The increased ED LOS during bundle implementation requires consideration of COVID-19 pandemic phenomena. The preimplementation period took place during pandemic onset when ED patient volumes were notably reduced, while the implementation period was associated with an increase in mental health visits and changes to ED flow processes.^51,52,53,54,55^ Thus, we did not study the bundle in the context of stable, underlying secular trends.^38,56^ In our study, ED LOS was compared between study periods with different throughput states.^57^ From 2020 to 2022, site EDs experienced a 16% average increase in annual visits overall, while mental and behavioral health ED visits increased by 20% at the Alberta Children’s Hospital. The bundle may have blunted what could have been even longer ED visits, which have been described by others during our implementation time period.^58^ As such, additional evaluation is needed to better understand the relationship of the bundle with LOS.

The associations we report for ED LOS and care satisfaction have implications for achieving optimal and equitable ED mental health care.^7^ While increased LOS was associated with reduced satisfaction, being seen by a mental health care provider or child and adolescent psychiatrist and hospitalization were associated with increased LOS and satisfaction. These findings suggest that there may be opportunities to improve efficiencies during mental and behavioral health visits (eg, time to consultation or admission) while simultaneously addressing expectations for care (eg, who provides ED mental health care). Additionally, our finding that several ethnic and racial backgrounds were associated with increased care satisfaction is different from previous reports^59,60^ and points to a need to learn more about ED care experiences and contributors to satisfaction among ethnic and racial groups to address potential care inequities.

Finally, it is notable that hospitalizations were lower in both EDs in the implementation period compared with the preimplementation period. Our finding is contrary to local trends before bundle implementation—at Alberta Children’s Hospital, ED mental health visits resulting in hospitalization had been increasing by 16% in 2020 and an additional 15% in 2021; at Stollery Children’s Hospital, these increases were 24% and 15%, respectively. Our finding is also contrary to the increase in hospitalization for pediatric mental health visits reported for other Canadian EDs during our implementation period.^55^ The reductions in hospitalization may be a result of the structured approach to informing clinical decision-making provided by the ASQ and HEADS-ED; alternatively or additionally, as has been previously reported,^61^ access to follow-up assessment has a powerful influence on admission decisions. That key clinical features—acuity, risk of self-harm, and symptoms that affect functioning (eg, depression)^62^—did not differ between the study periods suggests that clinical need was relatively similar across periods, and physician decision-making may have changed with tool use.

### Limitations

There are several limitations of note. First, while well-being scores can be important measures of overall mental health, other outcomes, such as distress, could have been selected to assess bundle outcomes. We focused on well-being at 30 days as this was the time point and outcome preferred by patient-partners; however, we recognize that the impact of the ED components of the bundle likely diminish with time. Second, due to resource limitations, we could not monitor urgent follow-up appointment attendance rates and postappointment referral patterns at both sites. Measuring these elements during the implementation period would have provided important information on the bundle. Additionally, we were unable to access coroner data on out-of-hospital deaths by suicide among children lost to follow-up within 30 days of the index visit to assess for unintended bundle consequences. Based on parent report, we are aware of 1 such death that occurred during the preimplementation period. No child deaths were noted for index or return ED visits.

## Conclusions

The mental health care bundle evaluated in this study aligned with AAP recommendations for providing optimal and equitable care to children seeking ED care for mental and behavioral health concerns. While the care bundle was not associated with higher child well-being 30 days after the ED visit, it was associated with higher well-being at 6 months and there were fewer hospitalizations during its delivery. While ED LOS increased during bundle implementation, this may have been due to changes in patient volumes and care processes that occurred in hospitals during the COVID-19 pandemic.

## Notes

Supplement 1.
Trial Protocol

Supplement 2. **eFigure 1.** Schematic Depicting Study Periods and Changes to ED Mental Health Care **eFigure 2.** Interrupted Time Series Graphs of Child Well-being During the Study **eTable 1. **A Summary of Baseline Well-Being Scores Across Participant Age Groups **eTable 2.** Multivariable Regression to Explore Bundle Effect on Child Well-Being Among Preadolescents Aged Less Than 13 Years **eTable 3.** Multivariable Regression to Explore Bundle Effect on Child Well-Being Among Adolescents Aged 13 to 17 Years

Supplement 3.
Nonauthor Collaborators

Supplement 4.
Data Sharing Statement
