# Supplementary material for: Outcomes Following a Mental Health Care Intervention for Children in the Emergency Department: A Nonrandomized Clinical Trial
Source: JAMA Netw Open. 2025 Feb 26;8(2):e2461972. doi: 10.1001/jamanetworkopen.2024.61972 (PMC11866027; doi:10.1001/jamanetworkopen.2024.61972)
Supplement: Supplement 2. — eFigure 1. Schematic Depicting Study Periods and Changes to ED Mental Health Care eFigure 2. Interrupted Time Series Graphs of Child Well-being During the Study eTable 1. A Summary of Baseline Well-Being Scores Across Participant Age Groups eTable 2. Multivariable Regression to Explore Bundle Effect on Child Well-Being Among Preadolescents Aged Less Than 13 Years eTable 3. Multivariable Regression to Explore Bundle Effect on Child Well-Being Among Adolescents Aged 13 to 17 Years [file jamanetwopen-e2461972-s002.pdf]

Supplemental Online Content

Newton AS, Thull-Freedman J, Xie J, et al. Outcomes following a mental health care intervention for children in the emergency department. *JAMA Netw Open*. 2025;8(2):e2461972. doi:10.1001/jamanetworkopen.2024.61972

eFigure 1. Schematic Depicting Study Periods and Changes to ED Mental Health Care

eFigure 2. Interrupted Time Series Graphs of Child Well-being During the Study

eTable 1. A Summary of Baseline Well-Being Scores Across Participant Age Groups

eTable 2. Multivariable Regression to Explore Bundle Effect on Child Well-Being Among Preadolescents Aged Less Than 13 Years

eTable 3. Multivariable Regression to Explore Bundle Effect on Child Well-Being Among Adolescents Aged 13 to 17 Years

This supplemental material has been provided by the authors to give readers additional information about their work.

© 2025 Newton AS et al. *JAMA Network Open*.

eFigure 1. Schematic Depicting Study Periods and Changes to ED Mental Health Care.

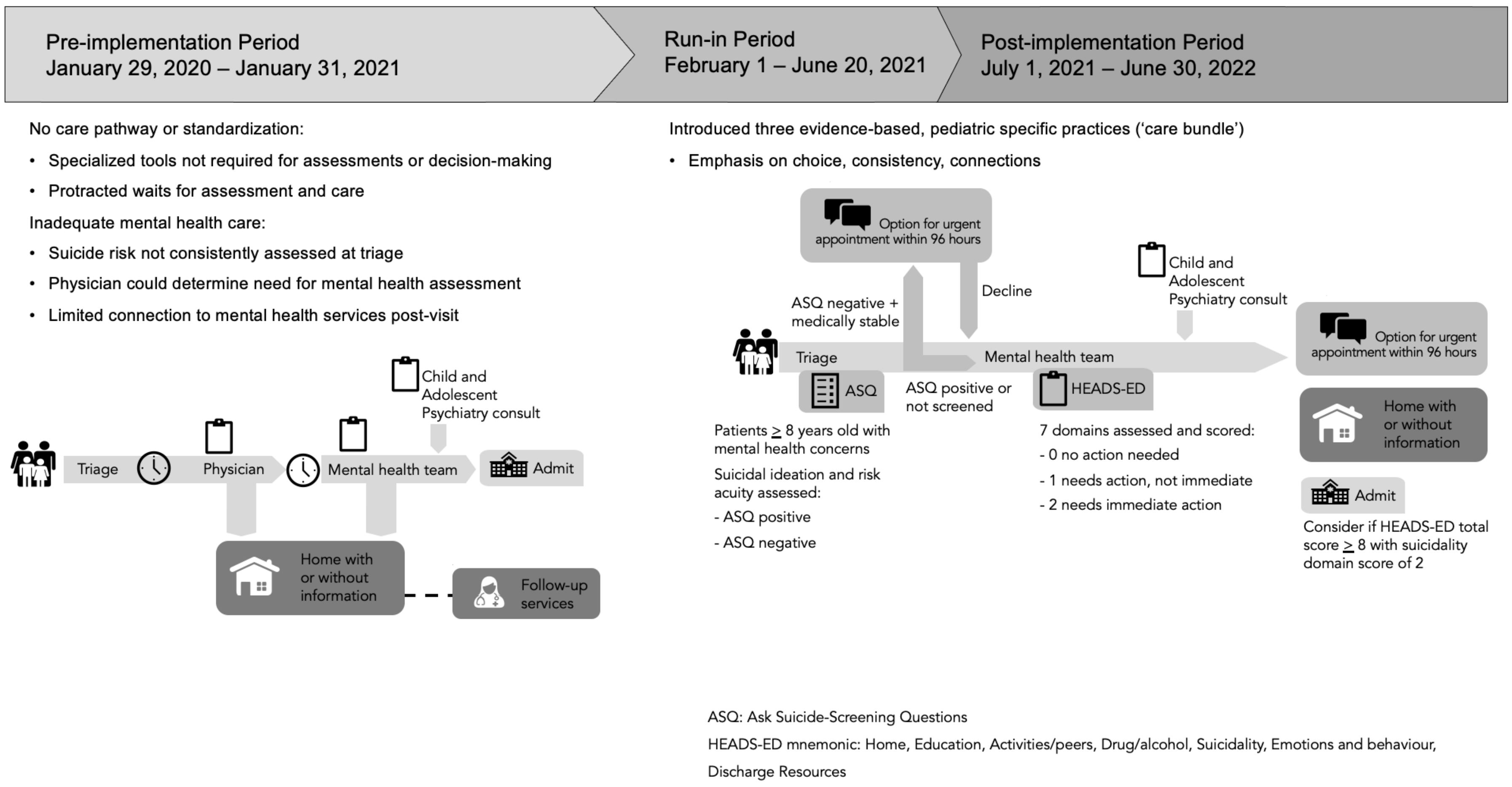

ASQ: Ask Suicide-Screening Questions; HEADS-ED mnemonic: Home, Education, Activities/peers, Drugs/alcohol, Suicidality, Emotions and behavior, Discharge Resources

© 2025 Newton AS et al. *JAMA Network Open*.

eFigure 2. Interrupted Time Series Graphs of Child Well-being During the Study.

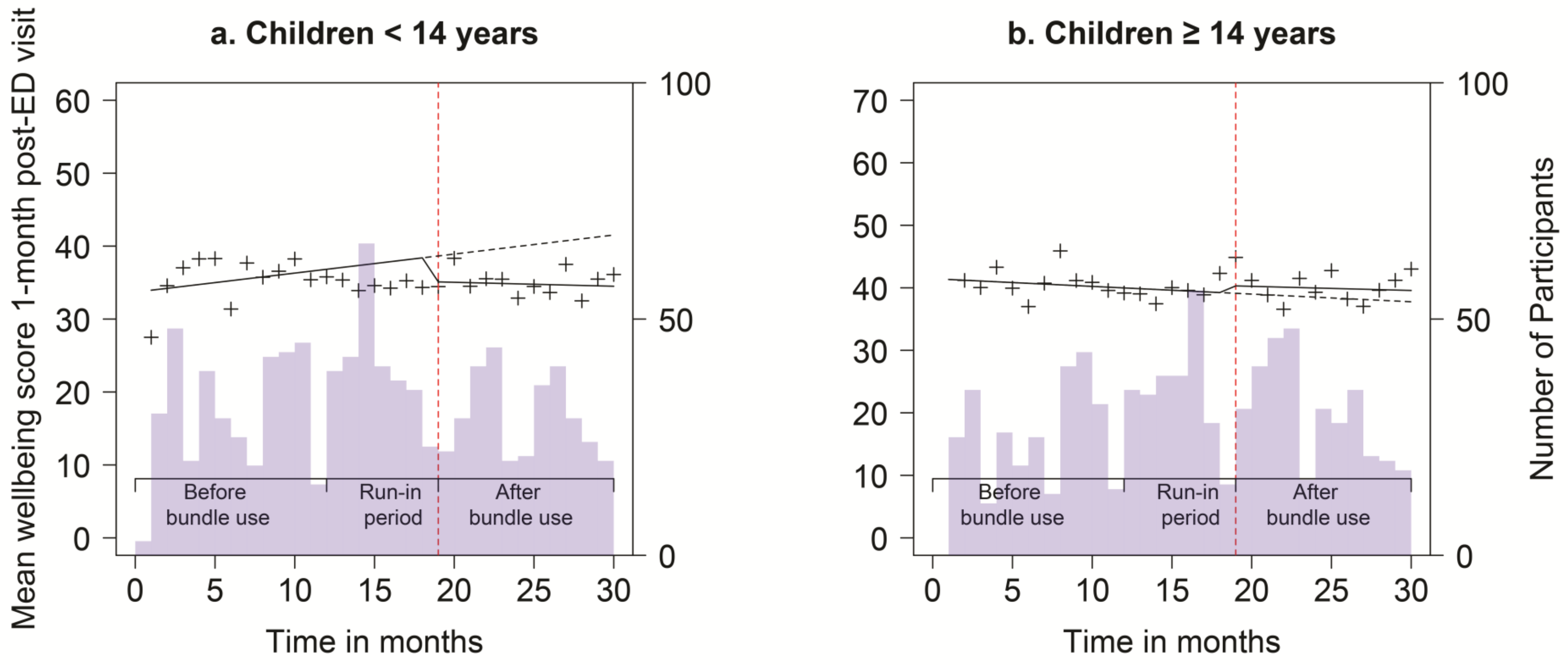

Footnotes:

The red vertical dashed line represents interruption; the black solid line represents trend lines of the outcome as well as post-intervention trend; the dashed black lines represent the counterfactual trend line. The barplots display the number of study participants.

Y-axis on the left side: well-being score; Y-axis on the right side: number of study participants. Figures a and b share the left and right Y-axis titles.

© 2025 Newton AS et al. *JAMA Network Open*.

eTable 1. A Summary of Baseline Well-being Scores Across Participant Age Groups.

Age group	Well-being Measure	Number in Analysis	Mean (SD)
**Before bundle implementation**			
3 to 5 years	Stirling Children's Well-Being Scale[a]	12	38.17 (8.95)
6 to 13 years	Stirling Children's Well-Being Scale[a]	346	33.52 (8.08)
14 to 17 years	Warwick-Edinburgh Mental Wellbeing Scale[b]	302	37.10 (8.87)
**After bundle implementation**			
3 to 5 years	Stirling Children's Well-Being Scale[a]	7	38.00 (6.43)
6 to 13 years	Stirling Children's Well-Being Scale[a]	310	33.25 (7.57)
14 to 17 years	Warwick-Edinburgh Mental Wellbeing Scale[b]	322	36.15 (7.998)

SD: standard deviation

[a] Score range: 12–60; [b] Score range: 14–70

© 2025 Newton AS et al. *JAMA Network Open*.

eTable 2. Multivariable Regression to Explore Bundle Effect on Child Well-Being Among Preadolescents Aged Less Than 13 Years.

	Child Well-being[a] Mean Difference (95%CI)	P value
**Age, per year older**	-0.04 (-0.08, -0.004)	0.033
**Gender identity**		
Male	0.02 (-0.13, 0.17)	0.803
Trans	0.05 (-0.85, 0.94)	0.921
Non-binary	-0.42 (-0.79, -0.06)	0.025
Not specified	-0.26 (-1.15, 0.61)	0.563
Female	Reference	
**Ethnoracial background**		
First Nations, Inuit, or Metis	-0.24 (-0.54, 0.06)	0.130
South, Southcentral, or Southeast Asian	0.40 (0.07, 0.72)	0.019
Black, Latin American, or West Asian[b]	0.29 (-0.10, 0.68)	0.148
Multiple	0.09 (-0.15, 0.34)	0.470
Not specified	0.61 (0.01, 1.21)	0.050
White	Reference	
**Visit acuity, per 1 CTAS category lower**	0.14 (0.03, 0.25)	0.014
**Diagnosis[c]**		
Suicidal ideation	-0.14 (-0.35, 0.07)	0.187
Neurotic, stress-related, and somatoform disorder	0.02 (-0.18, 0.23)	0.833
Mood disorder	-0.22 (-0.46, 0.02)	0.075
Behavioral or emotional disorder	-0.13 (-0.36, 0.10)	0.290
Self-harm not requiring medical care	-0.53 (-1.01, -0.04)	0.039
Any other assigned diagnosis[b]	-0.02 (-0.23, 0.18)	0.824
**Timing of well-being assessment**		
6 months post-visit	0.51 (0.42, 0.60)	<0.0001
3 months post-visit	0.79 (0.69, 0.90)	<0.0001

© 2025 Newton AS et al. *JAMA Network Open*.

30 days post-visit	0.27 (0.18, 0.36)	<0.0001
Baseline	Reference	
**Study period**		
Implementation	-0.03 (-0.17, 0.12)	0.737
Pre-implementation	Reference	

[a]z-score; [b] Groups combined due to sample size; [c] The reference group is without the individual specific diagnosis
Footnote: Interaction terms between time and implementation were not significant in the model and thus were not retained.

© 2025 Newton AS et al. *JAMA Network Open*.

eTable 3. Multivariable Regression to Explore Bundle Effect on Child Well-Being Among Adolescents Aged 13 to 17 Years.

	Child Well-being[a] Mean Difference (95%CI)	P value
**Age, per year older**	0.02 (-0.03, 0.06)	0.410
**Gender identity**		
Male	0.17 (0.04, 0.31)	0.014
Trans	0.14 (-0.15, 0.43)	0.361
Non-binary	-0.37 (-0.64, -0.10)	0.008
Not specified	-0.05 (-0.52, 0.43)	0.849
Female	Reference	
**Ethnoracial background**		
First Nations, Inuit, or Metis	0.12 (-0.10, 0.34)	0.275
South, Southcentral, or Southeast Asian	0.24 (-0.01, 0.49)	0.060
Black, Latin American, or West Asian[b]	0.26 (-0.03, 0.55)	0.084
Multiple	0.07 (-0.16, 0.31)	0.548
Not specified	-0.06 (-0.55, 0.43)	0.799
White	Reference	
**Visit acuity, per 1 CTAS category lower**	0.10 (0.01, 0.20)	0.034
**Diagnosis[c]**		
Suicidal ideation	-0.04 (-0.18, 0.11)	0.605
Neurotic, stress-related, and somatoform disorder	0.14 (-0.02, 0.29)	0.091
Mood disorder	-0.10 (-0.25, 0.05)	0.167
Behavioral or emotional disorder	0.17 (-0.19, 0.54)	0.368
Self-harm not requiring medical care	0.01 (-0.26, 0.29)	0.940
Any other assigned diagnosis[b]	0.02 (-0.15, 0.21)	0.786
**Timing of well-being assessment**		
6 months post-visit	0.72 (0.63, 0.81)	<0.0001
3 months post-visit	0.65 (0.57, 0.74)	<0.0001

© 2025 Newton AS et al. *JAMA Network Open*.

30 days post-visit	0.40 (0.33, 0.48)	<0.0001
Baseline	Reference	
**Study period**		
Implementation	0.01 (-0.11, 0.13)	0.883
Pre-implementation	Reference	

[a]z-score; [b] Groups combined due to sample size; [c] The reference group is without the individual specific diagnosis
Footnote: Interaction terms between time and implementation were not significant in the model and thus were not retained.

© 2025 Newton AS et al. *JAMA Network Open*.
