# Supplementary material for: Outcomes Following a Mental Health Care Intervention for Children in the Emergency Department: A Nonrandomized Clinical Trial
Source: JAMA Netw Open. 2025 Feb 26;8(2):e2461972. doi: 10.1001/jamanetworkopen.2024.61972 (PMC11866027; doi:10.1001/jamanetworkopen.2024.61972)
Supplement: Supplement 3. — Nonauthor Collaborators [file jamanetwopen-e2461972-s003.pdf]

Supplemental Online Content: Nonauthor Collaborators
*First name, last name, and suffix (if applicable) are required and will appear in PubMed.

*Group Name(s): Pediatric Emergency Research Canada (PERC)							
***First Name and Middle Initial(s)**	***Last Name**	***Suffix (eg, Jr, III)**	Academic Degrees	Institution	Location (city, state/province, country)	Role or Contribution, eg, chair, principal investigator	Group (if more than 1 Group listed in the byline) and/or Subgroup (eg, Steering Committee)
Samina	Ali		MDCM	University of Alberta	Edmonton, Alberta, Canada	Chair	
Waleed	Alquarashi		MSc, MD	Children's Hospital of Eastern Ontario	Ottawa, Ontario, Canada	Executive Board Member	
Brett	Burstein		MD, PhD	McGill University	Montreal, Quebec, Canada	Executive Board Member	
Tyrus	Crawford		BSocSc	Children's Hospital of Eastern Ontario	Ottawa, Ontario, Canada	Research Coordinator Representative	
Andrea	Eaton		MSc, BScN	University of Alberta	Edmonton, Alberta, Canada	National Cooredinator	
Gabrielle	Freire		MSc, MD	University of Toronto	Toronto, Ontario, Canada	Executive Board Member	
Michelle	Fric		MD	Stollery Children's Hospital	Edmonton, Alberta, Canada	Fellow Representative	
Naveen	Poonai		MSc, MD	Western University	London, Ontario, Canada	Vice Chair	
Bruce	Wright		MD	University of Alberta	Edmonton, Alberta, Canada	Executive Board Member	
Roger	Zemek		MD	University of Ottawa	Ottawa, Ontario, Canada	Past Chair	
