# Supplementary material for: Outcomes Following a Mental Health Care Intervention for Children in the Emergency Department: A Nonrandomized Clinical Trial
Source: JAMA Netw Open. 2025 Feb 26;8(2):e2461972. doi: 10.1001/jamanetworkopen.2024.61972 (PMC11866027; doi:10.1001/jamanetworkopen.2024.61972)
Supplement: Supplement 4. — Data Sharing Statement [file jamanetwopen-e2461972-s004.pdf]

Data Sharing Statement

Newton. Outcomes Following a Mental Health Care Intervention for Children in the Emergency Department. *JAMA Netw Open*. Published February 26, 2025. doi:10.1001/jamanetworkopen.2024.61972

Data
Additional Information: Clinicaltrials.gov, https://clinicaltrials.gov/study/NCT04292379, NCT04292379
Data available: No

Additional Information
Explanation for why data not available: The study was not approved to provide individual patient data outside of study team members.
